# Prevention of Contrast-Induced Nephropathy through a Knowledge of Its Pathogenesis and Risk Factors

**DOI:** 10.1155/2014/823169

**Published:** 2014-11-30

**Authors:** Michele Andreucci, Teresa Faga, Antonio Pisani, Massimo Sabbatini, Domenico Russo, Ashour Michael

**Affiliations:** ^1^Nephrology Unit, Department of Health Sciences, Magna Graecia University, Salvatore Venuta Campus, Viale Europa, Loc. Germaneto, 88100 Catanzaro, Italy; ^2^Nephrology Unit, Department of Public Health, University of Naples Federico II, Via Pansini No. 5, 80131 Naples, Italy

## Abstract

Contrast-induced nephropathy (CIN) is an iatrogenic acute renal failure (ARF) occurring after the intravascular injection of iodinated radiographic contrast media. During the past several years, in many patients undergoing computed tomography, iodinated contrast media have not been used for the fear of ARF, thereby compromising the diagnostic procedure. But recent studies have demonstrated that CIN is rarely occurring in patients with normal renal function and that preexisting chronic renal failure and/or diabetes mellitus represent(s) predisposing condition(s) for its occurrence. After the description of CIN and its epidemiology and pathophysiology, underlying the important role played by dehydration and salt depletion, precautions for prevention of CIN are listed, suggested, and discussed. Maximum priority has to be given to adequate hydration and volume expansion prior to radiographic procedures. Other important precautions include the need for monitoring renal function before, during, and after contrast media injection, discontinuation of potentially nephrotoxic drugs, use of either iodixanol or iopamidol at the lowest dosage possible, and administration of antioxidants. A long list of references is provided that will enable readers a deep evaluation of the topic.

## 1. Introduction

Contrast-induced nephropathy (CIN), which is also called contrast-induced acute kidney injury (CI-AKI), is an iatrogenic disease occurring after the intravascular injection of iodinated radiographic contrast media. CIN was first described in a patient with multiple myeloma receiving intravenous pyelography [1]. Today, the common opinion is that multiple myeloma* per se* cannot be considered a main risk factor for developing acute kidney injury following intravascular administration of iodinated contrast media [2]. In 2004 Gleeson and Bulugahapitiya [3] indicated CIN as the third leading cause of hospital-acquired acute renal failure (ARF) after surgery and hypotension, being responsible for 12% of all cases of ARF in hospital.

Meinel et al. [4] have recently underlined (a) that after modern iodinated radiographic media had been introduced in clinical practice they have been considered responsible for ARF [5], (b) that numerous subsequent noncontrolled observational studies appeared to confirm the causal role of contrast media for most cases of ARF following their intravascular administration [6], and (c) that, consequently, for many patients undergoing computed tomography (CT) iodinated contrast media have not been used for the fear of ARF, thereby compromising the diagnostic procedure [7]. Katzberg and Newhouse [8] have challenged this concept particularly for intravenous (i.v.) injection of iodinated contrast media.

Thus, the logical question that the clinicians ask themselves is whether CIN is still a clinical problem.

## 2. Contrast-Induced Nephropathy

CIN may be defined as an ARF that occurs within 24–72 hrs of exposure to i.v. or intra-arterial iodinated contrast media that cannot be attributed to other causes. In most cases it is a nonoliguric ARF with an asymptomatic transient decline in renal function, so that it may go undetected by those clinicians who do not check the renal function in the days following the contrast administration, as it is the case in nonhospitalized patients. The renal function impairment is mirrored by an absolute increase by 0.5 mg/dL (or greater) or relative increase by 25% (or greater) of serum creatinine from baseline or, better, by a decrease to 30–60 mL/min (renal insufficiency) or less in the estimated glomerular filtration rate (eGFR), that is, the creatinine clearance calculated using the MDRD (modification of diet in renal disease) formula [9] or the CKD-EPI (chronic kidney disease epidemiology collaboration) equation [10], or the very simple Cockcroft-Gault formula [11]. The rise in serum creatinine is peaking on the third to fifth day, returning to baseline within 10–14 days [12].

In some cases, CIN may cause a more severe impairment of renal function with oliguria (<400 mL/24 hrs), requiring dialysis. In these cases the mortality is high.

The clinical feature and the management of CIN are the same as that for ARF due to other causes [13–15].

## 3. Incidence of CIN

The early literature had greatly overestimated the incidence of CIN [16]. CIN occurs in up to 5% of hospitalized patients who exhibit normal renal function prior to the injection of contrast medium [17] and in about 2% [18] or even 1% of outpatients with eGFR > 45 mL/min per 1.73 m^2^ [19].

Thus, CIN is uncommon in patients with normal preexisting renal function. Actually, it occurs more frequently in patients with renal impairment, particularly if associated with diabetic nephropathy [8]. Among all procedures utilizing contrast agents for either diagnostic or therapeutic purposes, coronary angiography and percutaneous coronary interventions are associated with the highest rates of CIN [20]. This is mainly related to (a) the intra-arterial injection, (b) the high dosage of the contrast used, and (c) the type of patients who are usually in advanced age, with one or more comorbid conditions, such as advanced vascular disease, severe long-standing hypertension, diabetes, and some renal function impairments [18].

In a retrospective study analyzing 11,588 patients undergoing CT either without contrast or with the low-osmolar contrast medium iohexol or the isoosmolar contrast medium iodixanol Bruce et al. [21] observed that the incidence of CIN in the low-osmolar contrast medium group was similar to that of the control group up to a serum creatinine level of 1.8 mg/dL; but serum creatinine above 1.8 mg/dL was associated with a higher incidence of CIN in the low-osmolar contrast medium group; there was no significant difference in the incidence of CIN between the isoosmolar contrast medium and the control groups for all baseline serum creatinine values.

Recently, McDonald et al. [22] carried out a retrospective study on 53,439 patients in whom serum creatinine was regularly checked. They evaluated the effects of i.v. iodinated contrast media exposure to the incidence of CIN: the incidence was not significantly different in contrast media group compared to control group. In a meta-analysis of controlled studies by the same group [23] in patients exposed to i.v. contrast media compared with patients undergoing an imaging examination without contrast media (control group), the incidence of CIN, dialysis, and death was similar in the contrast media group and in the control group. They concluded that i.v. iodinated contrast media may not be the causative agent in diminished renal function after contrast material administration.

In an unselected, prospective, consecutive population of outpatients who received the low-osmolar, nonionic contrast iopamidol-370 for a contrast-enhanced CT study in the emergency department of a large, academic, tertiary care center Mitchell et al. [24] found an incidence of CIN of 11% (70 out of 633) of the patients enrolled; six of the 70 cases subsequently developed severe renal failure, five of whom required dialysis or died.

In a retrospective study performed over a 10-year period in 20,242 adult inpatients (10,121 untreated and 10,121 treated with i.v. iodinated contrast media) Davenport et al. [25] found that i.v. low-osmolality iodinated contrast media is a risk factor for nephrotoxicity in patients with a stable eGFR < 30 mL/min/1.73 m^2^. No nephrotoxicity was observed in patients with a pretomography eGFR > 45 mL/min/1.73 m^2^. The authors concluded that i.v. contrast medium is a nephrotoxic risk factor but not in patients with a stable serum creatinine <1.5 mg/dL or eGFR > 45 mL/min/1.73 m^2^ [26].

Rudnick and Feldman [27] have evaluated whether CIN is causally related to mortality and to what extent could mortality in patients undergoing contrast procedures be reduced by preventing CIN. After reviewing observational studies and clinical trials, they concluded that the deaths of some patients with CIN are complicated by factors that cannot be directly related to the use of contrast media, such as liver disease, sepsis, respiratory failure, and bleeding. On the other hand, patients undergoing coronary diagnostic procedures may already have some renal problems [28] even without using contrast media. It is, however, plausible that CIN contributes to cardiovascular causes of death in patients with CIN [27].

Concerning the long term outcome of patients who have had CIN, Solomon et al. [29] have studied in 294 patients, with follow-up of at least 1 year after contrast exposure, the relationship of CIN to long-term adverse events, such as death, stroke, myocardial infarction, end-stage kidney disease, percutaneous coronary revascularization, coronary artery bypass graft surgery, cardiac arrest, development of congestive heart failure or pulmonary edema, and the need for permanent pacing. The rate of these long-term adverse events was higher in individuals who had had CIN.

Permanent severe renal failure requiring dialysis occurs in 10% of patients with preexisting renal failure who develop further reduction in renal function after coronary angiography [30].

## 4. Pathophysiology of CIN

The intravascular injection of iodinated radiographic contrast media is followed by an immediate haemodynamic renal biphasic response: firstly a rapid and short renal vasodilatation with an increase in renal blood flow (RBF) followed by a prolonged vasoconstriction with an increase in intrarenal vascular resistances and a reduction in total RBF. The extrarenal vessels undergo a transient vasoconstriction followed by a stable decrease in vascular peripheral resistances [31].

**Figure 1:**
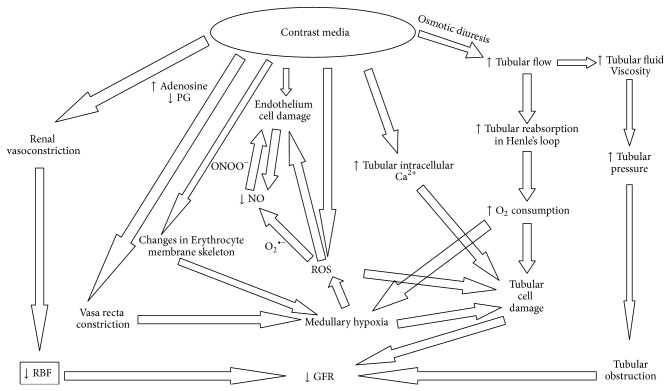
The complex mechanisms by which iodinated radiographic contrast media cause the fall of GFR.

These haemodynamic changes cause a decrease of glomerular filtration rate (GFR) and a renal ischaemia particularly in the renal medulla. The outer medulla, even under normal physiological conditions, receives little oxygen (O_2_) (because of its distance from the descending* vasa recta*) despite its high local oxygen consumption due to the important active tubular reabsorption in S3 segments of proximal renal tubules and in the medullary thick ascending limb of the Henle's loops that are located here. Prostaglandins, nitric oxide (NO), and adenosine continuously adjust medullary tubular transport activity to the limited available O_2_ supply, by enhancing the regional blood flow and downregulating the tubular transport [32]. Defects in one or more of these protective mechanisms will cause medullary hypoxia. The haemodynamic changes induced by contrast media will make medullary hypoxia quite severe (Figure 1).

However, radiographic contrast media also induce an osmotic diuresis that increases fluid delivery and consequent tubular reabsorption in the ascending limb of Henle's loops, thereby increasing energy need and O_2_ consumption: the result will be a worsening of medullary hypoxia [33–35] (Figure 1).

Medullary hypoxia causes the formation of reactive oxygen species (ROS) [36–38] that may exert direct tubular and vascular endothelial injury and might further intensify renal parenchymal hypoxia by virtue of endothelial dysfunction [39, 40] (Figure 1).

ROS cause a decrease in NO [36, 41]. The reaction between the ROS superoxide anion and nitric oxide leads to the formation of the more powerful oxidant peroxynitrite [42] which may cause more damage to the endothelial cells.

The injection of iodinated contrast media increases ROS production and renal oxidative stress which, in turn, mediates the damage to cell membranes leading to cellular apoptosis and necrosis, particularly in medullary thick ascending limbs and in S3 segments of proximal renal tubules of the outer medulla [36].

Recently, Pisani et al. [43] have demonstrated that a recombinant manganese superoxide dismutase administered* in vivo* to rats undergoing diatrizoate treatment was able to reduce renal oxidative stress, thereby preventing the reduction of GFR and the renal histological damage that follows contrast media administration.

Furthermore, iodinated contrast media also possess a direct cytotoxic property on endothelial and renal tubular cells, leading to apoptosis and necrosis [44]. The intravascular injection of contrast agents causes a direct endothelial damage that has been seen by scanning electron microscopy: cell shrinkage, nuclear protrusion, fenestration of the endothelial layer and formation of microvilli (“blebbing”) on the cell membrane, and cellular apoptosis [45]. The damaged endothelial cells contribute to the decrease in NO in the* vasa recta* [41] (Figure 1).

After the intravascular injection the iodinated contrast media are filtered by the glomeruli and are concentrated within the renal tubules (because of tubular fluid reabsorption), thereby exposing the renal tubular cells to a direct damage, which have been observed in studies of isolated tubular segments and cultured cells substantiated by disruption of cell integrity and apoptosis [46, 47].

The biochemical changes in the damaged epithelial tubular cells have been studied by evaluating the changes in major intracellular signalling pathways involved in cell survival, death, and inflammation [38, 48–52]* in vitro* in cultured renal tubular cells [53]. These aspects have been clarified in primary human tubular cells as well as in HK-2 cells exposed to different contrast media. A decrease of cell viability, secondary to a reduced activation/phosphorylation of Akt and of ERK 1/2, have been demonstrated, which was alleviated by transfecting the HK-2 cells with a constitutively active form of Akt [54]. In HK-2 cells, oxidative stress causes an increase in phosphorylation of Akt [50, 53] contrary to the effect of contrast media. In HK-2 cells contrast media affect the activation/deactivation of transcription factors, like FoxO3a and STAT3, that control the genes involved in apoptosis and cell proliferation [54–56].* In vivo* animal studies as well as* in vitro* studies suggest that iodinated contrast media can directly induce caspase-mediated apoptosis of renal tubular cells [57]. Contrast-induced apoptosis may also be due to the activation of shock proteins and the concurrent inhibition of cytoprotective enzymes and prostaglandins [58, 59].

In physiological conditions, the Na^+^/Ca^2+^ exchanger (NCX) is pumping the Ca^2+^ outside the renal tubular epithelial cells by using the Na^+^ concentration gradient across the cell membrane; this process is keeping low the intracellular Ca^2+^. Under pathological conditions, such as in CIN, NCX can reversibly extrude Na^+^ for Ca^2+^ influx thereby leading to intracellular Ca^2+^ overload. Intracellular Ca^2+^ overload is considered to be a key factor in ischemic cell injury and in CIN [60–62].

We have mentioned that the concentration of the contrast medium increases considerably within the tubular lumen because of tubular fluid reabsorption. Thus, the tubular fluid osmolality increases. Because of the exponential concentration-viscosity relationship, an overproportional increase in tubular fluid viscosity occurs [63]. Since the fluid flow rate through a tube increases with the pressure gradient and decreases with the flow resistance and since the resistance increases proportionally to fluid viscosity, the increased viscosity caused by a contrast medium increases the intratubular pressure [63]. Thus, the osmotic diuresis caused by the contrast media raises the intratubular pressure with a condition of tubular obstruction that contributes to the tubular epithelial damage and to the fall of GFR [35] (Figure 1).

The effect on morphology of erythrocytes by contrast media has also been studied [64, 65], with formation of echinocytes and stomatocytes observed upon incubation of erythrocytes with contrast media which may have a negative effect on the rheology of the blood [41]. More recent work has shown that contrast media affect the membrane skeleton of erythrocytes, with iopromide causing drastic changes in the band3-spectrin network compared with iodixanol that may contribute to microcirculatory disorders (especially in patients with coronary artery disease) and gas transport, contributing to tissue hypo-oxygenation [66].

## 5. The Iodinated Radiographic Contrast Media

The iodinated radiographic contrast media have different osmolalities and viscosities. The ionic High-Osmolar Contrast Media (HOCM, e.g. diatrizoate) have an osmolality of 1500 to 1800 mOsm/kg, that is, 5–8 times the osmolality of plasma. Nonionic Low-Osmolar Contrast Media (LOCM e.g., iohexol) have an osmolality of 600 to 850 mOsm/kg, that is, 2-3 times the osmolality of plasma. Nonionic isoosmolar contrast media (IOCM, e.g., iodixanol) have an osmolality of approximately 290 mOsm/kg, that is, the same osmolality as plasma [12, 35, 67].

Heinrich et al. [47] compared the cytotoxic effects of dimeric and monomeric iodinated contrast media on renal tubular cells* in vitro*. Results of this study indicated that HOCM have a greater potential for cytotoxic effects on proximal tubular cells* in vitro* than do LOCM or IOCM. At equal iodine concentrations (300 mg I/mL), the HOCM ioxithalamate showed stronger cytotoxic effects than did other contrast media. It has been demonstrated that the use of LOCM rather than HOCM is beneficial in reducing the incidence of CIN in patients with preexisting renal failure [68–71]. Adverse reactions to contrast media with the occurrence of CIN range from 5% to 12% for HOCM and from 1% to 3% for LOCM. Thus, the HOCM are used less frequently. There is no difference in the cytotoxicity of LOCM iomeprol and IOCM iodixanol at equal iodine concentrations in renal proximal tubular cells* in vitro* [72]. Recent studies and meta-analyses have shown no significant difference in the rates of CIN between IOCM and LOCM [72–75]; only the LOCM iohexol seems to be more nephrotoxic [68, 76]. The IOCM iodixanol seems less nephrotoxic than the LOCM iohexol, at least in patients with intra-arterial administration of the drug and renal insufficiency [72, 77].

Iodinated radiographic contrast media have also a different viscosity. The low osmolality achieved with the IOCM has come at the price of considerably increased viscosity; at comparable iodine concentrations and X-ray attenuation, the nonionic dimeric IOCM have about twice the viscosity of nonionic monomeric LOCM [63, 78, 79].

## 6. Preexisting Impairment of Renal Function and Diabetes Mellitus

CIN occurs more frequently in subjects with renal insufficiency, irrespective of cause. An eGFR of 60 mL/min/1.73 m^2^ is a reliable cut-off point for identifying patients at high risk for the development of CIN [12]. The higher the baseline creatinine value is or, better, the lower the eGFR is, the greater the risk of CIN is. Patients with chronic renal failure (CRF) have defective antioxidant systems [80] and increased oxidative stress associated with inflammation and endothelial dysfunction [81]. This may explain why preexisting renal failure represents the most common condition predisposing to the development of CIN [35].

Since clinical need for diagnostic and therapeutic procedures using contrast media (such as coronary angiography and percutaneous coronary interventions) is increased particularly in patients with cardiovascular diseases whose renal function is frequently impaired, the occurrence of renal damage by contrast media is quite frequent.

Diabetes mellitus is another predisposing factor to CIN, particularly when associated with impairment of renal function [82, 83]. At any given degree of baseline GFR, diabetes doubles the risk of developing CIN compared with nondiabetic patients. The incidence of CIN in diabetic patients varies from 5.7 to 29.4% [20]. Many authors do not regard the presence of diabetes mellitus in the absence of renal failure as a risk factor for CIN. In diabetic patients with preserved renal function and without other risk factors, in fact, the incidence of CIN has appeared comparable to that of a nondiabetic population [84]. Coupling chronic kidney disease and diabetes dramatically increases the risk for CIN compared with that observed for chronic kidney disease alone [85].

Furthermore, the effects of diabetes and prediabetes on the development of CIN have been investigated by Toprak et al. [86] on 421 patients with chronic kidney disease undergoing coronary angiography; 137 had diabetes mellitus, 140 had prediabetes and 144 had a normal fasting glucose; CIN occurred in 20% (RR = 3.6), 11% (RR = 2.1), and 5.5%, respectively. Haemodialysis was required in 3.6% and 0.7% of those with diabetes and prediabetes, respectively. A serum glucose concentration above 124 mg/dL (6.8 mmol/l) was the best cut-off point for prediction of CIN.

The biologically active endothelins, produced by proteolysis of the precursor prepro-endothelins under the action of endothelin-converting enzyme, are increased in circulating blood of diabetics. In diabetic patients there is also a hypersensitivity of renal vessels to adenosine [87, 88]. These factors may justify the predisposition of diabetics to the development of CIN [34].

## 7. Additional Predisposing Factors

The European Society of Urogenital Radiology has stated that the real risks for CIN are represented by preexisting renal impairment particularly when secondary to diabetic nephropathy, but also to salt depletion and dehydration, congestive heart failure, an age greater than 70 years, and concurrent use of nephrotoxic drugs [89, 90].

Salt depletion and dehydration deserve a special discussion. Dehydration is the decrease of body water. This, for instance, is what occurs in old patients who do not force themselves to drink water during the day, due to impaired sensation of thirst [91]. But the term dehydration is frequently used to indicate salt and water depletion with contraction of extracellular volume. Anyhow, dehydration and salt depletion are responsible for the reduction of “effective” intravascular volume [92]. The “effective” circulating blood volume may be defined as the relative fullness of the arterial tree as determined by cardiac output, peripheral vascular resistance, and total blood volume [14]. A reduction of the “effective” circulating blood volume may be due to congestive heart failure, compromised left ventricle systolic performance, prolonged hypotension or liver cirrhosis or nephrotic syndrome or salt depletion [92].

When we perform intravascular injection of contrast media in patients who are dehydrated and/or hypovolemic, water and salt overreabsorption occurs in renal tubules causing a further increase of the intratubular concentration of contrast material and, due to the concentration-viscosity relationship, overproportionally increasing tubular fluid viscosity. This is why dehydration and/or volume contraction (salt depletion following abnormal gastrointestinal, renal or dermal fluid losses associated with insufficient salt intake and reduction of “effective” circulating blood volume [93]) are major risk factors for CIN. Thus, prehydration and correction of volume depletion are very important in all patients before any diagnostic and therapeutic procedures requiring intravascular injection of contrast media [94].

Advanced age [20, 95], anemia [96], severe congestive heart failure, or compromised left ventricle systolic performance [20], sepsis [3, 95, 97], and renal transplant [98] represent additional risk factors for CIN.

In an interesting study of Ranucci et al. [99] the risk of postoperative acute renal insufficiency after cardiac surgery in 423 adults was increased by the volume of administered radiocontrast medium and was more likely when there was a short interval between angiography and subsequent cardiac surgery. The authors suggested that cardiac surgery should be delayed beyond 24 hours of exposure to contrast agents when feasible and that the use of these agents should be minimized.

The concomitant use of nephrotoxic drugs, such as aminoglycosides, cyclosporine A, amphotericin, cisplatin, dipyridamole, and nonsteroidal anti-inflammatory drugs, is undoubtedly another factor favoring CIN. The authors of many studies believe that patients with CRF under treatment with angiotensin-converting enzyme inhibitors (ACEIs) or angiotensin II receptor blockers (ARBs) are at high risk for developing CIN [100–106], particularly in the elderly [107]. According to KDIGO (kidney disease improving global outcomes) guidelines for Acute Kidney Injury Work Group, there is insufficient evidence to recommend discontinuation of these medications prior to contrast administration [108].

## 8. Precautions for Prevention of CIN

Thus, CIN is not as frequent as it was believed to be in the past few years. However, it does occur in high risk patients. This makes it necessary to use all precautions that may prevent contrast media-nephrotoxicity [12, 109–112].

The first precaution is that, in patients undergoing a radiographic procedure, the renal function should be monitored by measuring serum creatinine before and once daily for 5 days after the contrast medium injection [3].

The second precaution is that potentially nephrotoxic drugs (aminoglycosides, vancomycin, amphotericin B, dipyridamole, metformin, and nonsteroidal anti-inflammatory drugs) should be discontinued before the radiographic procedure [12]. If aminoglycosides are necessary, it is suggested to follow the European Renal Best Practice [113] recommendation: “Do not use more than one shot of aminoglycosides for the treatment of infections… In patients with normal kidney function in steady state, aminoglycosides are administered as a single-dose daily rather than multiple-dose… monitoring aminoglycoside drug levels.”

Special attention should be paid to the use of metformin, an oral antihyperglycemic drug used to treat type II diabetes that stimulates intestinal production of lactic acid. Metformin is excreted unchanged almost entirely by the kidneys; thus, it is retained in cases of CIN and may cause a severe lactic acidosis that can be fatal. Thus, this medication should be discontinued 12 hours before the administration of contrast agent and not be resumed until at least 36 hours after the procedure, or even longer if the serum creatinine has not returned to baseline [114].

The third precaution is an adequate hydration of the patient [115, 116]. Not only should the old suggestion to limit fluid intake starting the day before contrast administration be abolished, but it is important to give an adequate supplement of water. It has been suggested to give the patient 500 mL of water orally before and 2,500 mL for 24 hours after contrast injection to secure urine output of at least 1 mL/min in a nondehydrated patient [117]. In high-risk patients it may be useful to implement, instead, the i.v. infusion of 0.9% saline at a rate of about 1 mL/kg b.w. per hour, beginning 6–12 hours before the procedure and continuing for up to 12–24 hours after the radiographic examination, provided that urine output is appropriate and the cardiovascular condition allows it [3, 115]. The increase of urine output that follows the hydration will limit the duration of contrast material contact with the epithelial cells of the renal tubules and consequently its cytotoxicity [118, 119]. Some investigators have obtained better results using sodium bicarbonate instead of sodium chloride [120–129]: 154-mEq/L infusion of sodium bicarbonate as a bolus of 3 mL/kg b.w./hour for 1 hour before the administration of contrast, followed by 1 mL/kg/hour for 6 hours during and after the procedure [121]. The increase of bicarbonate excretion would decrease the urine acidification, thereby reducing the production and increasing the neutralization of oxygen free radicals [123, 124, 130–132]. Other investigators did not find any benefit with sodium bicarbonate versus sodium chloride [133–136]. The European Renal Best Practice [113] “recommends volume expansion with either isotonic sodium chloride or sodium bicarbonate solutions, rather than no volume expansion, in patients at increased risk for CIN.”

The fourth precaution is choosing the least nephrotoxic iodinated agent. As mentioned above, iodixanol (IOCM) and iopamidol (LOCM) appear to be contrast agents of choice to reduce risk of CIN [73].

The fifth precaution is the use of contrast media at the lowest dosage possible. The use of large doses and multiple injections within 72 hrs [3, 95, 137], in fact, represent risks for CIN that are dose-dependent [138–142]. High doses of contrast agents are required in coronary angiography and percutaneous coronary interventions. Fortunately, the development of newer imaging technologies has facilitated faster image acquisition; this has enabled radiologists to perform studies with less intravascular contrast, because the duration of time over which contrast needs to be administered has shortened [12, 143]. Anyhow for these procedures some formulas have been suggested to calculate the dosage that is least dangerous for renal function: (a) Cigarroa's formula: 5 mL of contrast per kg b.w./serum creatinine (mg/dL) with maximum dose acceptable of 300 mL for diagnostic coronary arteriography [144]; (b) Laskey's formula: volume of contrast to eGFR ratio with a cut-off point of the ratio at 3.7 for percutaneous coronary intervention [145]; recently the cut-off point has been placed at 2.0: below a ratio of 2.0 CIN may be a rare complication of percutaneous coronary intervention, which would increase dramatically at a ratio of 3.0 [143, 146]; (c) a ratio of the grams of iodine to eGFR; a ratio of 1.42, or even better a ratio of 1.0, would prevent CIN [143].

## 9. Use of Antioxidants for Preventing CIN

We have seen that ROS play an important role in the nephrotoxicity by iodinated radiocontrast agents. Thus, it has been thought that antioxidants could be useful in preventing CIN. N-acetylcysteine has been the first tested antioxidant, considering also its double properties, as a free-radical scavenger as well as a drug able to increase the vasodilating effect of NO [3, 39, 147]. Lee et al. [148] treated human embryonic kidney cells with three different contrast media: the ionic HOCM ioxithalamate, the nonionic LOCM iopromide, and the IOCM iodixanol; all three contrast media caused a significant reduction of cell viability at 24 hours (*P* < 0.001); short-duration pretreatment with N-acetylcysteine significantly improved cell viability compared with no N-acetylcysteine pretreatment (*P* < 0.001). Despite controversial results observed in high risk patients [13, 149–158], it has been suggested to use N-acetylcysteine in high-risk patients either with an oral dose of 600 mg twice daily the day before and the day of the procedure [3] or with an i.v. dose of 150 mg/kg half an hour before the procedure or 50 mg/kg administered for 4 hours [151].

Conflicting results have been obtained with the use of the antioxidant ascorbic acid. Thus, some authors have demonstrated that prophylactic oral administration of ascorbic acid may protect against contrast-induced CIN [159–161] at a dosage of 3 g orally 2 hours before the procedure and 2 g during the night and in the morning after the procedure [159, 160]. Other authors demonstrated a nonprotective effect [162]. In a recent meta-analysis, with 1536 patients who completed the trial, patients receiving ascorbic acid had a 33% less risk of developing CIN [161].

Three different methods for preventing CIN have been interestingly compared some years ago by Briguori et al. [163] in 326 patients with chronic kidney disease: 0.9% saline infusion + N-acetylcysteine (*n* = 111), sodium bicarbonate infusion + N-acetylcysteine (*n* = 108), and 0.9% saline + ascorbic acid + N-acetylcysteine (*n* = 107). The mean amounts of contrast medium (iodixanol) administered were 179+/−102, 169+/−92, and 169+/−94 mL (*P* = 0.69), respectively; and risk scores (9.1+/−3.4, 9.5+/−3.6, and 9.3+/−3.6; *P* = 0.21) were similar in the three groups. CIN occurred in 11 of 111 patients (9.9%) after saline + N-acetylcysteine, in 2 of 108 (1.9%) after bicarbonate + N-acetylcysteine, and in 11 of 107 (10%) after saline + ascorbic acid + N-acetylcysteine. The authors concluded that sodium bicarbonate + N-acetylcysteine was superior to the other two methods.

In another study by Poletti et al. [164], out of 87 adults with renal insufficiency who underwent emergency CT scanning, 43 were hydrated and given N-acetylcysteine (900 mg) intravenously; the other 44 were only hydrated. There was a 25% or greater increase in serum creatinine concentration in two of the former and in nine of the latter. However, there was a 25% or greater increase in serum cystatin C concentration in seven and nine patients respectively. This disjunction between the effects of acetylcysteine on creatinine and cystatin levels led the authors to suggest that acetylcysteine might prevent the rise in serum creatinine after contrast administration without actually preventing CIN.

Tasanarong et al. [165] carried out a prospective, double-blind, randomized and placebo-controlled trial in 305 patients with CRF undergoing coronary procedures with iopromide (LOCM). The oral administration of 2 antioxidants, either 350 mg/day of *α*-tocopherol or 300 mg/day of *γ*-tocopherol (5 days prior to the procedure and continued for a further 2 days post-procedure) in combination with 0.9% saline (1 mL/kg/h for 12 hours before and 12 hours after) was shown to protect against CIN: CIN occurred in 14.9% of cases in the placebo group, but only in 4.9% and 5.9% in the *α*- and *γ*-tocopherol groups, respectively.

Nebivolol, a third-generation *β*_1_-adrenergic receptor antagonist, has been suggested for protecting kidney against CIN because its antioxidant and NO-mediated vasodilating action [166–168]: 5 mg/day for one week or 5 mg every 24 hours for 4 days have been shown to decrease the incidence of CIN in patients with renal dysfunction undergoing coronary angiography [169, 170].

Recent studies have shown a beneficial effect of statins in preventing CIN in patients undergoing percutaneous coronary intervention [171–176]. The nephroprotective effect of statins has been attributed to their antioxidant, anti-inflammatory, and antithrombotic properties and to their vasodilator property mediated by NO that improves renal microcirculation [177, 178]. Rosuvastatin, at a dosage of 10 mg/day for five days, administered two days before and three days post the radiographic procedure, reduced the risk of CIN in patients with diabetes mellitus and CRF undergoing coronary/peripheral arterial angiography [179]. In patients with acute coronary syndrome, scheduled for an early invasive procedure, rosuvastatin, at a dosage of 40 mg on admission followed by 20 mg/day, has been shown to reduce the incidence of CIN [180]. Short-term atorvastatin (40 mg/day 3 days before the procedure) and chronic atorvastatin therapy had a protective effect on renal function after coronary angiography [181]. Short-term high-dose atorvastatin (80 mg 12 hours before intervention with another 40 mg preprocedure dose) has been shown to decrease the incidence of CIN in patients with acute coronary syndrome undergoing percutaneous coronary interventions [182]. These results suggest the early use of high-dose statins before percutaneous coronary revascularization to protect patients against contrast media nephrotoxicity.

## 10. Other Protective Measures

As mentioned above, enhanced transport activity in the outer renal medulla will increase oxygen consumption, thereby causing renal hypoxia, whose role is important in the pathogenesis of CIN. Thus, furosemide has been suggested for protecting the kidney by reducing the active tubular reabsorption. Furthermore, the consequent increase in urine output will decrease the contact time of contrast material with tubular epithelium, thereby reducing the epithelial damage. But the increase of urinary salt excretion with the diuretic may cause salt depletion in the absence of adequate fluid replacement. Thus, Marenzi et al. [183] have suggested the perfect combination of hydration plus furosemide: this was obtained by delivering i.v. fluid in an amount exactly matched to the volume of urine produced by the patient under the effect of furosemide: the result was a significantly lower incidence of CIN when compared to the patients treated with hydration only.

As mentioned above, the intracellular Ca^2+^ overload is considered to be a key factor in ischemic cell injury and in CIN. The increase in intracellular calcium provokes a vasoconstrictive response in intrarenal circulation and would be an important mediator of epithelial cell apoptosis and necrosis. Thus, calcium channel blockers have been hypothesized to have protective effects against CIN. Their use, however, has given controversial results, some authors suggesting them to be protective [184, 185], whilst others finding no benefit at all [44, 186–188].

Since urinary adenosine is increased after contrast media administration, it has been thought that adenosine antagonists, such as theophylline or aminophylline, could have protective effects against contrast media. Similarly also for these drugs their use has given controversial results, with some finding beneficial effects against CIN [189–192], whilst others finding no beneficial results [193, 194].

Plasma and urine levels of endothelin-1 are increased in diabetes and after exposure to high doses of contrast media; this has suggested a role of endothelin-1 in diabetic nephropathy and in CIN [87, 195, 196]. However, endothelin receptor blockers have been proven to be deleterious as a prophylactic tool against CIN [197].

Trimetazidine (TMZ) has been described as a cellular anti-ischemic agent [198]. Previous studies demonstrated that TMZ prevents the deleterious effects of ischaemia-reperfusion at both the cellular and the mitochondrial levels, and exerts potent antioxidant activity on various tissue preparations [199, 200]. TMZ inhibits the excessive release of oxygen-free radicals, increases glucose metabolism, limits intracellular acidosis, protects ATP stores, reduces membrane lipid peroxidation and inhibits neutrophil infiltration after ischaemia-reperfusion. Onbasili et al. [201] studied the efficacy of TMZ in the prevention of CIN in 82 patients with high serum creatinine concentration undergoing coronary angiography/angioplasty. TMZ (20 mg thrice daily) was given orally for 72 hours starting 48 hours before the procedure and all the patients were given intravenous isotonic saline (1 mL/kg) for 24 hours starting 12 hours beforehand. CIN developed in only one of 40 patients who were given TMZ (2.5%) and in seven of 42 controls (17%). The authors concluded that the administration of TMZ (20 mg thrice daily, orally) in conjunction with saline is an effective means for preventing transient renal dysfunction due to radiocontrast agent; they admitted several limitations of their study, such as the relatively small sample size investigated and the dose of oral TMZ that had been chosen for this study that was derived from the standard regimen for the treatment of myocardial ischaemia. In addition, relatively low-risk patients with mild renal insufficiency had been included in that study. The effect of TMZ on high-risk patients should be further investigated in larger prospective clinical studies to determine whether it can be useful for significantly preventing CIN.

Dopamine and dopamine agonists, such as fenoldopam, a selective dopamine-1 receptor agonist with vasodilatory properties, have given controversial results in protecting against CIN, some positive [202–204], others negative [153, 194, 205–207]. On the basis of our present knowledge, it is better to avoid these drugs because of their adverse effects (arrhythmia with dopamine, and systemic hypotension with intravenous fenoldopam).

Recombinant human erythropoietin (EPO; 200 U/mL) has been also used by Kolyada et al. [208] in order to investigate the protection of renal tubular cells against contrast medium-induced injury* in vitro* in LLC-PK1 (a well-known non-human renal tubular epithelial cell line), which were exposed to a non-ionic low-osmolar agent iohexol and to the isoosmolar agent iodixanol for 6 hours: EPO improved the viability of the iohexol-treated cells by 27% and the viability of the iodixanol-treated cells by 26%. It also reduced apoptosis rates and attenuated activation of caspase-3, caspase-8, and caspase-9.

Previously, Goldfarb et al. [209] had demonstrated that EPO pretreatment prevented renal dysfunction in a rat model of CIN, induced by iothalamate. EPO was given to male Sprague-Dawley rats (EPO group) at the dosage of 3000 U/kg and 600 U/kg, 24 and 2 h before the induction of CIN, respectively. The decline in creatinine clearance in control (CTR group; subjected to saline) animals was prevented by EPO pre-treatment. The extent of medullary thick ascending limb- and S3-tubular damage in the outer renal medulla, however, was comparable in the two experimental groups (“CTR” and “EPO”).

In another study by Yokomaku et al. [210], asialoerythropoietin, a nonhemopoietic derivative of erythropoietin, attenuated contrast-induced renal dysfunction and renal tubular damage in rats, an effect that was attributed to inhibition of apoptosis by activation of Janus kinase 2 (JAK2) and the phospho-JAK2/signal transducer and activator of transcription 5 (STAT5).

No clinical studies are available at the moment to confirm these experimental data in humans by demonstrating a protective role of EPO in patients subjected to CIN.

## 11. Haemodialysis and Hemofiltration

It has been suggested to remove radiocontrast media by hemodialysis or hemofiltration immediately after the radiographic procedure. Schindler et al. [211] demonstrated, in patients with CRF (most of whom in chronic dialysis), that different dialysis techniques do remove contrast media (iopromide or iomeprol), high-flux hemodialysis and hemodiafiltration more effectively than low-flux hemodialysis and hemofiltration. But Lehnert et al. [212] demonstrated that, although hemodialysis eliminates contrast media, it does not prevent CIN. Vogt et al. [213] performed a randomized trial to test whether CIN can be avoided by prophylactic hemodialysis immediately after the administration of low-osmolality contrast media in patients with impaired renal function (baseline serum creatinine level >2.3 mg/dL); renal function was recorded before and during the 6 days after administration of contrast media. The prophylactic hemodialysis did not diminish the rate of CIN. These results suggested that, even if dialysis is carried out immediately, the early damage has already triggered a cascade of pathogenic events, which cannot be reversed [214, 215].

It is worthwhile to point out that the European Renal Best Practice [113] does “not recommend using prophylactic intermittent hemodialysis or hemofiltration for the purpose of prevention of CIN.”

## 12. Conclusion

To answer the initial question as to whether CIN is still a clinical problem, we may underline that today the incidence of CIN is less than it was in the past, provided that clinicians identify the patients at risk and use all the precautions listed above, particularly monitoring renal function before, during, and after the radiographic procedure and preventing dehydration and salt depletion. However, CIN is still too frequent in patients at high risk for contrast nephrotoxicity because of concomitant diseases such as diabetes mellitus with renal failure, severe congestive heart failure, reduction of “effective” circulating blood volume, sepsis. We do hope to significantly reduce the occurrence of CIN also in these patients with the development of newer and less toxic iodinated contrast media.

## Abbreviations

CIN: Contrast-Induced nephropathy
CI-AKI: Contrast-induced acute kidney injury
ARF: Acute renal failure
CT: Computed tomography
i.v.: Intravenous
MDRD: Modification of diet in renal disease
CKD-EPI: Chronic kidney disease epidemiology collaboration
eGFR: Estimated glomerular filtration rate
RBF: Renal blood flow
GFR: Glomerular filtration rate
NO: Nitric oxide
O_2_: Oxygen
ROS: Reactive oxygen species
HOCM: High-osmolar contrast media
LOCM: Low-osmolar contrast media
IOCM: Iso-osmolar contrast media
CRF: Chronic renal failure
ACEIs: Angiotensin-converting enzyme inhibitors
ARBs: Angiotensin II receptor blockers
EPO: Erythropoietin
TMZ: Trimetazidine.
